# The Significance of Mesophilic *Aeromonas* spp. in Minimally Processed Ready-to-Eat Seafood

**DOI:** 10.3390/microorganisms7030091

**Published:** 2019-03-23

**Authors:** Sunniva Hoel, Olav Vadstein, Anita N. Jakobsen

**Affiliations:** Department of Biotechnology and Food Science, NTNU–Norwegian University of Science and Technology, N-7491 Trondheim, Norway; olav.vadstein@ntnu.no (O.V.); anita.n.jakobsen@ntnu.no (A.N.J.)

**Keywords:** *Aeromonas*, ready-to-eat, seafood, sushi, sashimi, food safety, food spoilage, virulence

## Abstract

Minimally processed and ready-to-eat (RTE) seafood products are gaining popularity because of their availability in retail stores and the consumers’ perception of convenience. Products that are subjected to mild processing and products that do not require additional heating prior to consumption are eaten by an increasing proportion of the population, including people that are more susceptible to foodborne disease. Worldwide, seafood is an important source of foodborne outbreaks, but the exact burden is not known. The increased interest in seafood products for raw consumption introduces new food safety issues that must be addressed by all actors in the food chain. Bacteria belonging to genus *Aeromonas* are ubiquitous in marine environments, and *Aeromonas* spp. has held the title “emerging foodborne pathogen” for more than a decade. Given its high prevalence in seafood and in vegetables included in many RTE seafood meals, the significance of *Aeromonas* as a potential foodborne pathogen and a food spoilage organism increases. Some *Aeromonas* spp. can grow relatively uninhibited in food during refrigeration under a broad range of pH and NaCl concentrations, and in various packaging atmospheres. Strains of several *Aeromonas* species have shown spoilage potential by the production of spoilage associated metabolites in various seafood products, but the knowledge on spoilage in cold water fish species is scarce. The question about the significance of *Aeromonas* spp. in RTE seafood products is challenged by the limited knowledge on how to identify the truly virulent strains. The limited information on clinically relevant strains is partly due to few registered outbreaks, and to the disputed role as a true foodborne pathogen. However, it is likely that illness caused by *Aeromonas* might go on undetected due to unreported cases and a lack of adequate identification schemes. A rather confusing taxonomy and inadequate biochemical tests for species identification has led to a biased focus towards some *Aeromonas* species. Over the last ten years, several housekeeping genes has replaced the 16S rRNA gene as suitable genetic markers for phylogenetic analysis. The result is a more clear and robust taxonomy and updated knowledge on the currently circulating environmental strains. Nevertheless, more knowledge on which factors that contribute to virulence and how to control the potential pathogenic strains of *Aeromonas* in perishable RTE seafood products are needed.

## 1. Introduction

Over the last several years, the assortment of lightly processed seafood products available in supermarkets and retail stores has grown tremendously. In compliance with the customers’ preferences for natural, healthy and convenient food, the consumption of minimally processed seafood that require minimal preparation has gained popularity [1,2]. Despite the general awareness of the positive health benefits from seafood, fish is not consumed as often as recommended—a tendency found more often among younger consumers in many countries [3,4]. This can largely be attributed to the fact that fish is perceived as relatively difficult to prepare [1,5]. This has been a driver for the development of an assortment of ready-to-eat (RTE) seafood products.

Minimal food processing aims to preserve the sensory and nutritional properties of the raw material to the greatest extent possible. Many minimally processed seafood products fall in to the RTE category, i.e., products that do not require heating before consumption, but would normally need refrigeration. To extend the shelf life of perishable RTE seafood, refrigeration combines with mild preservation methods such as salt, smoke, fermentation, vacuum packaging, or modified atmosphere packaging. Mild processing may not completely prevent growth of microorganisms and applies to a range of seafood products, including marinated mussels, shrimps, pasteurized crustaceans, smoked fish products and raw fish based products such as sashimi grade fish loins, sushi and seafood salads. When hurdle technology is bypassed or reduced, the need for high standard production practices becomes more significant. For instance, temperature control during shelf life is of utmost importance for controlling microbial growth in perishable seafood, but it may vary throughout the value chain [6,7]. When heating is not performed prior to consumption, an important barrier against the transfer of pathogenic microorganisms is lost. As a result, the consumption of raw or undercooked seafood can cause a number of diseases.

Worldwide, seafood is an important source of foodborne outbreaks. In the EU, seafood accounted for more than 10% of strong evidence outbreaks in 2015, but the total burden of seafood-associated disease is probably larger owing to unreported cases [8]. When ingestion is accounted for, seafood has the highest risk for foodborne disease among all major food groups, and this situation is largely accounted for by the increased interest in RTE seafood in industrialized countries [9]. According to the CSPI Outbreak Alert (Center for Science in the Public Interest) [10], naturally occurring biotoxins such as scomrotoxin and ciguatoxin in finfish, and pathogens such as *Vibrio* and norovirus in shellfish, are the major causes of seafood mediated diseases. *Vibrio* infections are increasing, and the infections are most often associated with the consumption of raw oysters or undercooked mussels [11]. In Japan, *V. parahaemolyticus* is one of the leading causes of foodborne gastroenteritis and annually, 500–800 outbreaks affecting more than 10,000 people are reported [12]. It is estimated that this pathogen accounts for half of the foodborne illnesses in Asian countries [13].

The current trend, where raw seafood such as sushi and sashimi (slices of raw fish) are consumed more frequently by an increasing part of the population, introduces new food safety concerns. The most susceptible groups for foodborne infections include children, pregnant women, older adults, and people with weakened immune systems [14]. The two latter groups constitute an increasing part of the population. Food that is normally safe for healthy adults can lead to foodborne illness for those in the more susceptible groups. Additionally, these groups are more likely to have a lengthier illness, undergo hospitalization, and develop life-threatening disorders [15]. The current EFSA (European Food Safety Authority) scientific report stated that *Listeria* cases increased among people over age 75 and among women aged 25–44 (believed to be pregnancy related) over the time period 2008–2015 [16]. The increased proportion of older adults in the population combined with the upswing in consumption of RTE food is believed to contribute to this trend.

Special attention must be paid to the pathogenic bacteria that can enumerate in perishable RTE seafood during refrigerated storage, such as *Listeria monocytogenes* and *Aeromonas* spp. In general, *L. monocytogenes* has a high tolerance towards low storage temperature (≤4 °C), and is recognized as one of the major microbiological hazards in seafood production. As a consequence, their presence in food production environments and RTE seafood products is thoroughly surveilled [16]. Bacteria belonging to the genus *Aeromonas* are ubiquitous aquatic bacteria, recognized as increasingly important human pathogens due to the frequent prevalence in all types of food, and particularly in all types of seafood. As more of the seafood is eaten raw, new food safety issues emerges and there is a need to identify the microbial hazards in new RTE seafood products. To date, the knowledge on the growth and disease-causing potential of *Aeromonas* spp. in minimally processed seafood and in seafood products intended for raw consumption is very limited. Only a few studies have highlighted the presence of this pathogen in lightly processed RTE seafood products such as sushi [17,18,19], cold smoked salmon [20], and molluscan shellfish [21]. This review aims to summarize the status of *Aeromonas* as a potential foodborne pathogen, and to discuss the significance of the presence of *Aeromonas* spp. in raw or minimally processed RTE seafood products on the retail market.

## 2. The RTE Seafood Trend from a Food Safety Perspective

The increasing interest in RTE seafood may, from a public health perspective, have a positive effect on the intake of fish and seafood, especially among the younger population. Due to the widespread availability in retail stores, products based on raw fish such as prepackaged sushi meals are consumed by an increasing proportion of the population [22,23]. However, changing eating habits result in new microbiological issues and emphasizes the need for public knowledge on safe handling and preparation of raw fish. “*Food safety is the assurance that food will not cause harm to the consumer when it is prepared and/or eaten according to its intended use*” ([24], p. 6). This implies that the responsibility for safe food is shared by all involved in the food chain, from production to final preparation and to consumption.

In general, the food safety of RTE products relies on numerous factors including raw material quality (ingenious microbiota), inherent food characteristics (e.g., water activity, NaCl, pH), processing technology, storage temperature, packaging (atmosphere), shelf-life (short or extended), production hygiene (food contact surfaces, hygienic design, slicing equipment and utensils, bare hand contact), and cross contamination between ingredients. Raw fish is an excellent substrate for bacterial growth due to the high *post mortem* pH and high levels of soluble nitrogen compounds in the tissue [25]. The fish is susceptible to contamination during processing operations such as filleting and slicing. In filter-feeding molluscan shellfish such as oysters, clams and mussels, pathogenic microorganisms can concentrate in the gut where they can multiply. Moreover, combined products such as assembled pieces of sushi and seafood salads are subjected to possible contamination from non-seafood ingredients, mainly vegetables. The natural contamination of raw vegetables is normally high due to the close association with soil, and there is a high risk for contamination through irrigation water [26]. RTE vegetables, including leafy vegetables, are often contaminated with a large microbial population, typically in the range 5 to 7 log CFU/g [27,28]. Washing of fresh produce with tap water is frequently applied in an attempt to remove soil and microorganisms. However, the method is not efficient for all types of fresh produce [29]. An ingredient-based survey of a retail sushi production revealed that washed and chopped vegetables (spring onion) had the highest concentration of bacteria compared to other sushi ingredients. The dominating bacteria were lactic acid bacteria (LAB), Enterobacteriaceae and *Aeromonas* spp., all of which could constitute a food spoilage or food safety problem in the assembled sushi during storage [19]. The microbiological complexity of such combined products with minimal processing highlights the need for additional hurdles to prevent cross-contamination from raw vegetables to the raw fish or other ingredients.

Pathogen contamination of RTE seafood is particularly hazardous when it takes place after processing, especially after the application of microbe-reducing processes. This reduces the microbial competition and the chance for some pathogens to outgrow increases thereafter [9]. Food handlers are important vehicles for contamination of microorganisms from human to food, and RTE food prepared manually are frequently linked to outbreaks of foodborne illness [30,31]. Use of gloves, appropriate hand washing, and quarantine of personnel showing clinical symptoms can contribute to prevention of spread of microorganisms with human reservoirs such as norovirus, *E. coli* and *Staphylococcus aureus*. Cross-contamination may also take place in the processing environment or during food preparation prior to consumption due to contact between raw material and contaminated surfaces and equipment, or from a contaminated food batch. The establishment of pathogenic bacteria in the processing environment can be a long-term source of contamination, as seen with *L. monocytogenes* in fish processing environments [32].

A number of biological hazards (which are not addressed in this review) are linked to seafood (Table 1). *V. parahaemolyticus* is the leading bacterial cause of seafood borne outbreaks linked to minimally processed or raw seafood. Oysters are often eaten raw, at least in the U.S., and they are one of the most common foods associated with *V. parahaemolyticus* infection [12]. Other outbreaks have been caused by bacterial pathogens such as *Clostridium botulinum*, *Staphylococcus aureus*, *Bacillus cereus*, *Shigella*, *Salmonella*, *and Aeromonas* [12,33,34,35,36]. A multistate outbreak of *Salmonella* infections linked to raw tuna in sushi resulted in 62 cases of disease and 11 hospitalizations [37]. Other agents, such as *L. monocytogenes* and *E. coli*, must also be considered as risk factors due to their presence in raw or lightly preserved seafood and vegetables [18,38,39,40,41]. *L. monocytogenes* is currently not reported as particularly prevalent in RTE seafood, such as retail sushi [19,42,43]. However, the prevalence of *Aeromonas* spp. in retail seafood is higher and may vary from six to 72% [44,45,46,47], and high prevalence (71 to 90%) was reported in retail sushi [18,19]. Significant seafood pathogens other than *Aeromonas* spp. were reviewed by Løvdal [48] and Elbashir et al. [49].

Among viruses, Norovirus is considered the major causative agent of seafood borne disease [50]. A variety of parasitic infections might be acquired by consumption of raw or undercooked seafood. Anisakiasis is a zoonosis caused by nematode parasites belonging to the genus *Anisakis*. The highest incidence of anisakiasis is found in Japan due to the high intake of raw seafood and the frequency is now rising in Western countries because of changes in eating habits [51]. This tendency is also observed for the fish tapeworm *Diphyllobothrium*, associated with freshwater fish or marine fish that spawn in freshwater rivers (such as wild salmon). Cases of this parasite have declined in previously endemic areas because of improved sanitary conditions, whereas the number of cases has increased in Western countries. The widespread popularity of sushi and other lightly processed seafood dishes are presumably a major contributor to this increase [52]. To prevent human disease from *Anisakis* spp. and other parasites, the current EU legislation [53] requires that all fish for raw consumption must be frozen to a core temperature of minus 20 °C for at least 24 hours. However, this regulation does not apply to farmed Atlantic salmon, as the risk of acquiring *Anisakis* nematodes is very low due to the use of formulated feed [54,55]. This was conferred in a recent Norwegian survey of more than 4000 whole, farmed salmon where no anisakids were present in the salmon intended for consumption [56].

The total global burden of foodborne disease is not known, mainly because of the lack of reports of all cases to public health authorities [11]. Moreover, it is reasonable to assume that a great proportion of affected people never seek medical care, and that the causative agent cannot be identified. In fact, a significant proportion of seafood-mediated illness is probably caused by a heterogeneous group of less understood agents. Estimates suggest that at least 38 million cases of foodborne illness in the U.S. are caused by unspecific agents each year [57]. Many agents are known to cause gastroenteritis, but their significance in illness cannot be estimated due to lack of data. This includes *Aeromonas* infections that are likely to go under the surveillance radar due to the disputed status as a true foodborne pathogen. Moreover, the lack of selective media for isolation from mixed samples can lead to misidentification of *Aeromonas* strains from clinical samples. *Aeromonas* spp. can grow on *Vibrio* isolation agars like TCBS (thiosulfate-citrate-bile-salt-sucrose agar) and thus be misidentified as being a *Vibrio* [58]. In the following, the taxonomy, virulence and prevalence of the bacteria belonging to genus *Aeromonas* are discussed.

## 3. Introduction to the Genus *Aeromonas*

The genus *Aeromonas* belongs to the Aeromonadaceae family and are Gram-negative, facultative anaerobic, oxidase-positive, catalase positive, rod-shaped bacteria. Species belonging to the genus *Aeromonas* can be classified into two main groups. The first group is composed of the psychrophilic, non-motile strains mainly infecting fish, and is primarily represented by *Aeromonas salmonicida*. The second and larger group is motile, mesophilic *Aeromonas* species, and many of them are associated with human disease [59,60]. The taxonomy of the genus *Aeromonas* is complex and has undergone numerous changes over the last two decades. Prior to 1985, only three species were known [59]. The exact number of species is constantly changing because of the frequent description of new species and reclassifications of already accepted species. In the most recent 2005 version of the Bergey’s Manual of Systematic Bacteriology, the number of species in the genus *Aeromonas* was 14, in addition to the previously so-called Enteric group 501, later classified as *Aeromonas diversa* [59,61]. The *Aeromonas* species were previously classified based on sequence analysis of the 16S rRNA gene and DNA-hybridization groups [62]. The implementation of protein-coding housekeeping genes as high-resolution phylogenetic markers has enabled the discovery of several new species over the past ten years and has led to a more clear and robust taxonomy. To date, 36 species of *Aeromonas* are recognized (Table 2), whereof 19 so far have been implicated in human disease and are considered human opportunistic pathogens [63].

## 4. Taxonomic Controversies and Reclassifications of Species in the Genus *Aeromonas*

Over the past two decades, the genus *Aeromonas* had been subjected to many deep phylogenetic analyses, as recently reviewed by Navarro and Martinez-Murcia [94]. The first phylogenetic analysis of the genus was based on sequencing of the 16s rRNA gene [95]. These analyses supported a majority of the previous classifications based on phenotypic traits, but it was the first evidence of the taxonomic complexity of this genus. The ambiguous taxonomy of the genus *Aeromonas* can be summarized into three main issues: (1) 16S rRNA sequencing cannot separate all known species, (2) species reclassifications and new species proposals based on rarely isolated species, and (3) a high rate of misidentification by biochemical tests.

Sequencing of the 16S rRNA gene has proven unsuccessful for identification of all *Aeromonas* species due to low taxonomic resolution [96,97]. The *Aeromonas* 16S rRNA gene sequences are extremely conserved when compared to other bacteria and lacks thus enough specificity for differentiating closely related species [94,98]. The interspecies similarity of the 16S rRNA gene sequences for the genus *Aeromonas* range from 96.8 to 100% [64]. According to Nagar et al. [97], the overall mean 16S rRNA sequence similarity for *Aeromonas* spp. was 97.3%, highlighting the poor discriminatory power of this genetic marker. Moreover, the interspecies similarity for the 16S rRNA sequences of a group of species particularly relevant for seafood, *A. salmonicida*, *Aeromonas bestiarum* and *Aeromonas piscicola*, was reported as high as 99.8–100%, and thus impossible to separate by 16S rRNA analysis [99]. For that reason, sequencing of housekeeping genes coding for proteins are now preferred as high-resolution single-gene phylogenetic markers with a focus on proteins related to DNA processing such as *gyrB* (encoding the B-subunit of DNA gyrase, a type II DNA topoisomerase) [100], and *rpoD* (encoding the σ^70^ factor conferring promoter specific initiation of transcription on RNA polymerase) [101]. The discriminatory power of the *gyrB* and *rpoD* genes are considerably higher than for the 16S rRNA gene, and mean sequence similarities for the housekeeping genes are in the range 89 to 93% [97,101,102]. Other single-gene phylogenetic markers applied for phylogenetic studies of *Aeromonas* are *rpoB* [103], *dnaJ* [104] and *recA* [105].

Caution is recommended when using a single housekeeping gene in phylogenetic analysis as they all do not have the same phylogenetic resolution or they might be affected by the process of horizontal gene transfer or events of genetic recombination [63,66,94]. For those reasons, it is advised to build phylogenetic trees on several housekeeping genes. While a single-gene strategy (mainly *gyrB*) has been used frequently to infer phylogenetic relationships [106,107,108], a borderline discriminatory power of *gyrB* was observed for the most similar species [100,102]. To overcome such limitations, multilocus phylogenetic analysis (MLPA) is considered appropriate to construct robust phylogenetic trees with non-overlapping levels of intra-species and inter-species genetic divergence [94,109].

The taxonomic confusions in the genus *Aeromonas* are further complicated by the frequent proposal of new species and the reclassification of recognized species. This can be explained by the organization of the genus in several species complexes, and new descriptions of species arrive from the most homogenous groups inside a complex. Consequently, new species are frequently described but some of them are subjected to controversies about their actual delineation [110]. For example, the description of *Aeromonas rivipollensis*, a new species related to *Aeromonas media*, is weak and in need of revision [110]. The clinically relevant *Aeromonas hydrophila* subsp. *dhakensis* [73] and *Aeromonas aquariorum* [72] were reclassified as *Aeromonas dhakensis* [74]. For decades, *A. dhakensis* has been mistaken for *A. hydrophila*, and this has probably led to an overestimation of the clinical significance of *A. hydrophila*. It is now evident that *A. dhakensis* is widely distributed in the environment (water and food) and in clinical samples, mainly in tropical and subtropical areas, and it must be recognized as a potential human pathogen [111,112,113]. *A. dhakensis* is thus an illustrative example of the importance of an accurate taxonomy for understanding the distribution and virulence potential of species belonging to the genus *Aeromonas*. Moreover, it is estimated that 30% of the nucleotide sequences deposited as *A. hydrophila* in nucleotide databases under the name *A. hydrophila* do not belong to this species [114]. Thus, there is reason to believe that an erroneous prevalence of *A. hydrophila* in food, water and clinical cases is reported. For instance, the US Food and Drug Administration consider only *A. hydrophila* as a food safety issue, even though many species may be more relevant [64].

Last, but not least, a significant proportion of the controversies of the genus *Aeromonas* may be attributed to the rather ambiguous species identifications based on biochemical tests. Conventional biochemical tests do not necessarily correspond to results achieved by genetic methods, and this is evident for environmental isolates of *Aeromonas* species [115,116]. Phenotypic identification systems can give misleading results because of the great variability in the responses. Some of the variability is probably due to differences in the running conditions of the biochemical tests, such as reaction temperature and incubation time. A set of *Aeromonas* strains isolated from Common carp (*Cyprinus carpio*) were identified as *A. hydrophila/Aeromonas caviae* by the API 20NE test system. Sequencing of the *rpoD* gene of these strains and subsequent phylogenetic analysis revealed that the isolates were *A. salmonicida* [98]. Moreover, in a study of clinical isolates from Danish stool samples, only 37% of the *Aeromonas* strains were correctly identified by the phenotypic method (a panel of biochemical tests) [117]. Comparisons of species assignation using genetic information versus various biochemical tests have revealed that the biochemical tests (commercial systems as well as panels of conventional biochemical tests) may misidentify up to 70% of the strains as being *A. hydrophila*. As a result, there is a biased overestimation of the clinical and environmental importance of *A. hydrophila*.

## 5. Is *Aeromonas* a True Foodborne Pathogen?

*Aeromonas* species, referred to as “a jack of all trades”, have received increasing attention as human pathogens lately. However, their classification as true foodborne pathogens continue to be debated. *Aeromonas* spp. are ubiquitous aquatic bacteria, widespread in different water sources and in food in general. They are readily isolated from meat, raw milk, poultry, fish, shellfish, and vegetables [19,38,113,118,119,120]. In many cases, the presence of *Aeromonas* spp. in food reflects food–water contact during harvest or processing. Water is considered the main source of food contamination, even though other reservoirs exists—for instance, animal feces, soil, chironomid egg masses and plankton [64,121].

There is a growing interest in the genus *Aeromonas* because of its pathogenic effects on aquatic organisms and humans, and its impact on food spoilage. A subset of four species is more implicated in human infections, and accounts for most of the clinical strains. These are *A. caviae*, *A. dhakensis*, *Aeromonas veronii* biovar *sobria* and *A. hydrophila* [122,123,124]. The diseases range from acute gastroenteritis to life-threatening conditions like septicemia and in rare cases, meningitis [125]. Water-associated skin and wound infections with aeromonads are commonly reported, and *Aeromonas* spp. were isolated as pure culture from 22% of the wounds of infected patients after the 2004 tsunami in Thailand [126]. The clinical manifestation of *Aeromonas*-mediated gastrointestinal disease associated with intake of contaminated food and water may vary considerably, from mild cases of self-limiting diarrhea to more severe cases that can result in a dysenteric form. Once established in the gastrointestinal tract, aeromonads can cause diarrhea by production of enterotoxins or by invasion of the gastrointestinal tract [127,128,129].

The role of *Aeromonas* as a true enteropathogen has been questioned due to few documented outbreaks and the low number of acute illnesses in a human challenge study [114]. Although *Aeromonas* spp. are isolated from clinical stool samples, their role as causative agent in gastrointestinal disease is still debated. This is further complicated by the fact that *Aeromonas* spp. has been isolated from stool samples of asymptomatic persons, and the carriage rate ranges from 3 to 30% in tropical and developing counties, respectively [130]. Others have reported a significantly higher incidence of *Aeromonas* spp. in patients with diarrheal disease compared to people considered as asymptomatic carriers [122]. As for other opportunistic microorganisms, susceptible patients include babies, young children, elderly, and patients with a preexisting illness including the immunocompromised [122,129]. There is a lot of inaccurate information regarding clinical isolates because of the widespread erroneous identification of the species. For that reason, the predominance of *A. hydrophila* concerning virulence, antibiotic resistance, and clinical characteristics should be regarded as unreliable.

Consumption of contaminated water and food are considered the main sources of human *Aeromonas* infections, but there are few reports of well-documented outbreaks that provide information on the ingested doses of *Aeromonas*. The only available human challenge study for *A. hydrophila* shows low susceptibility for development of acute symptoms, with only 2 out of 57 (3.5%) challenged persons affected after exposure to 10^10^ cells of strains isolated from clinical sources [131]. However, it is difficult to draw conclusions regarding infective doses based on a single human challenge study. Records from *Aeromonas* mediated outbreaks in Norway and Sweden suggest an infective dose in the range 10^6^ to 10^8^ cells [132], although the infective dose in some cases were lower (10^3^ to 10^4^ cells) [133]. There are clear links between cases of diarrhea and consumption of *Aeromonas* through drinking water despite a limited number of reported outbreaks [134,135,136,137] or through consumption of contaminated food [132,138]. By comparing the genotypes of water-borne and clinical isolates of *Aeromonas,* Khajanchi and colleagues [134] observed successful colonization and infection by particular strains after transmission from water to humans. Strong evidence of *Aeromonas* outbreak was attributed to the consumption of contaminated well water [139], and the outbreak of acute gastroenteritis affecting 33 people in Bhutan (South Asia) was linked to the consumption of carcass cow meat contaminated with *A. hydrophila* [140]. The exact species assignation in the latter study must be considered as tentative, as the identification was based on biochemical tests.

The risk of acute gastrointestinal disease linked to the concentration of *A. hydrophila* present in different water and food matrices was simulated using data from the human challenge study and data from selected outbreaks where the dose and numbers of affected persons were known [114]. The concentration of *A. hydrophila* in a cold dish identified as the source of a substantial Chinese outbreak affecting 349 people [138] was estimated to 2.3–3.0 log CFU/g [114]. Based on the simulations, the authors suggested that *A.s hydrophila*, and possibly other *Aeromonas* species, is highly infectious and that the potential to cause disease might be similar to undisputed enteropathogens like *Campylobacter* and *Salmonella.* The current knowledge from clinical studies and recent outbreaks confirms that *Aeromonas* should be treated as a human enteropathogen. Depending on host–microbe interactions, exposure to low to moderate doses of pathogenic *Aeromonas* strains may lead to infections, and it is fair to assume that many cases remain undiagnosed. Clearly, there is a need for thorough characterization of foodborne isolates for a better understanding of the link between host, environment and bacterial factors that lead to clinical symptoms and illness.

## 6. How to Reveal the Bad Guys—The Multifactorial Virulence of *Aeromonas*

The bad guys must have virulence factors. However, there are still many unresolved questions regarding the role that each of the virulence factors plays in *Aeromonas*-mediated gastroenteritis. The rather ambiguous clinical picture of the infections and the lack of a definite link between intake and illness indicate complex pathogenic mechanisms. The current knowledge indicates that the virulence of *Aeromonas* species is multifactorial. Many virulence factors involved in infection are described for different *Aeromonas* species and they can be classified into structural components, extracellular proteins, and parts of secretion systems. Virulence factors such as adhesins, flagella, toxins, and various exoenzymes have been identified in *A. hydrophila*, *A. dhakensis*, *A. salmonicida*, *A. jandaei*, *A. veronii*, and *A. caviae* among others [124,125,141]. The known *Aeromonas* virulence mechanisms were summarized by Tomás [123] and da Silva et al. [106]. They include, in brief: (1) fimbriae, flagella, and capsule allowing attachment to the host surface, (2) toxins and enzymes such as proteases, elastases, and hemolysins causing cell and tissue damage, (3) secretion systems enabling evasion of the host immune response, (4) iron binding proteins (siderophores) scavenging iron from the host, (5) capsule, S-layer, lipopolysaccharide, and porins compromising host defenses, (6) biofilm formation allowing adherence to cell surfaces, and (7) quorum sensing systems involved in regulation of virulence gene expression. Production and secretion of virulence factors such as adhesins, cytotoxins, hemolysins, lipases and proteases, and the ability to form biofilm by specific pathways that mediate expression of virulence through quorum sensing are demonstrated in *A. hydrophila* [142,143].

The virulence-associated extracellular proteins that have received the most attention are the cytotoxic enterotoxin Act (encoded by *act*), toxins with hemolytic activity, aerolysin (encoded by *aerA*) and hemolysin (encoded by *hlyA*), and the cytotonic enterotoxins Ast and Alt (encoded by *ast* and *alt*, respectively). One well-characterized hemolytic toxin is the β-hemolysin of *Aeromonas*, often referred to as aerolysin [144]. The gene encoding this pore-forming toxin (*aerA*) was isolated from foodborne isolates, including *A. hydrophila*, *A. dhakensis*, *A. salmonicida*, *A. bestiarum*, *A. media*, and *A. piscicola*, and with lower prevalence in *A. caviae* [102]. Ørmen and Østensvik [145] demonstrated that the prevalence of *aerA* varied with species among strains isolated from water. The species most often implicated in gastrointestinal infections, *A. veronii* biovar *sobria*, *A. hydrophila*, and *A. caviae* were *aerA* positive most frequently. A second family of β-hemolysins, the *Aeromonas* hemolysin (HlyA), also widespread in various *Aeromonas* spp., exhibits significant amino sequence homology to the HlyA hemolysin in *Vibrio cholerae* [146]. The genes encoding aerolysin and hemolysin are suggested to comprise a two-component system in which both genes must be inactive to reduce virulence [128]. A third *Aeromonas* enterotoxin with hemolytic activity is the cytotoxic enterotoxin Act, a type II pore-forming enterotoxin [127]. It is recognized that Act induces accumulation of fluid in the intestines and stimulates pro-inflammatory responses by increased cytokine production through elevated concentrations of tumor necrosis factor, Interleukin-1β and Interleukin-6 [147], and thereby plays an important role in *Aeromonas* infections. Moreover, Act is described as the main pathogenic factor of *A. hydrophila* responsible for hemolytic, cytotoxic and enterotoxic activities [123], and the toxin was lethal when injected to mice [148]. For a group of non-hemolytic foodborne strains of *A. media* and *A. caviae*, the lack of hemolytic activity was possibly related to the absence of the *act* gene [102]. Other toxins that are believed to have a role in *Aeromonas*-induced gastrointestinal disease are the *Aeromonas* heat-labile cytotonic enterotoxin, Alt, and a heat-stable cytotonic enterotoxin, Ast [127]. Regardless of geographical origin, the *alt* and *ast* genes seem to be less prevalent than *act* in food‒ and waterborne isolates [18,113,149]. However, *alt* and *ast* were highly prevalent in isolates from raw seafood [102], although their exact role in pathogenesis is not known. Various secretion systems are believed to contribute significantly to virulence in the complex pathogenicity of *Aeromonas* spp. The conserved type II secretion system (T2SS) is widely present in *A. hydrophila* [142] and it secretes Act. Act is considered a potent virulence factor in the *A. dhakensis* SUU strain, with the ability to cause both diarrhea and severe tissue damage in the host [141,150]. The type III secretion system (T3SS) is believed to act as a needle, and it is frequently found in clinical isolates [127,151], and in stains causing fish furunculosis [152]. A functional T3SS and the effector proteins AexT and AeuX have previously been identified in *A. hydrophila* [153]. Contact between the bacteria and the host epithelial cells was fundamental for the cytotoxicity of clinical *Aeromonas* isolates in vitro, and the cytotoxic activity of the strains was strongly associated with the presence of T3SS [154].

The existing knowledge about many *Aeromonas* virulence factors is based on analyses of the *A. hydrophila* AH-3 and SSU strains, which later were reclassified as *A. piscicola* AH-3 [82] and *A. dhakensis* SSU [74]. Clearly, there is lack of knowledge on the link between virulence factors and their distribution in various *Aeromonas* species. Various strains can possess multiple virulence factors in different combinations [106,155]. Some studies have reported that clinical isolates possess virulence factors more frequently than environmental strains [106,145]. Others have found no differences regarding the presence of virulence genes among clinical and environmental strains [155,156,157]. Furthermore, *Aeromonas* strains isolated from a variety of food products were shown to harbor a high number of virulence genes similar to those of clinical isolates [102,155,158]. Heterogeneity in the distribution of virulence genes among food isolates, also within species, indicate that the pathogenic potential may be strain‒ rather than species‒related. On the contrary, and of particular interest, is isolates belonging to *A. caviae* that seem to have lower prevalence of the known virulence genes compared to other species [60,102,124,155,159,160]. Grim et al. [141] demonstrated that the *act* gene was absent from all *A. caviae* isolates (clinical and water isolates). *A. caviae* is considered one of the most potent human pathogens in the genus *Aeromonas,* highly clinical relevant, and frequently associated with infections [115,135]. This indicates that the virulence of *A. caviae* involve other virulence factors than those mentioned above.

*A. dhakensis* SSU (formerly known as *A. hydrophila*) is one of the most studied clinical isolates. It encodes at least four distinct factors with enterotoxic properties in vitro; *hlyA*, *act*, *ast*, and *alt* [161]. We do not know the exact role of these factors in diarrhea, or if this combination of genes is representative for the pathogenic properties of diarrheic strains. The presence of genes encoding a type VI secretion system, aerolysin, hemolysin, Ast, and lateral flagella were the characteristics of an *A. trota* stain isolated from cerebrospinal fluid of a meningitis patient. This isolate also exhibited swarming motility, hemolytic activity, and adhesion and cytotoxicity in vitro [125]. A complicating issue is that some of the genes, for example *act*, are found in species not considered to be of high clinical relevance, such as *A. bestiarum*, *A. piscicola*, and *A. salmonicida.* Moreover, the *act* gene was highly prevalent in environmental strains isolated from drinking water (70%) [162] and from seafood (75–86%) [102,113]. Less virulent strains may have reduced expression of the gene [141].

Human *Aeromonas* infections have occasionally been followed by the life-threatening complication hemolytic-uremic syndrome (HUS) [163]. It is estimated that up to 50% of patients who develop HUS require renal dialysis, and the mortality rate is particularly high for children [164]. HUS is mainly associated with gastrointestinal infections produced by Shiga-toxin producing *E. coli* (the most common serotype is O157:H7) [165]. The Shiga-toxins are encoded by *stx* genes in the genome of a lysogenic bacteriophage (Stx phage) and represent a horizontal transfer mechanism [166]. The *stx-1* and *stx-2* genes have previously been detected in clinical and environmental strains of *A. hydrophila*, *A. caviae* and *A. veronii* biovar *sobria* [166,167,168]. Studies of clinical isolates in Mexico indicate that the *stx* genes are putative virulence factors of *Aeromonas*. In more detail, the *stx1* gene alone was detected in 33% of isolates, and the *stx1* and *stx2* genes together were detected in 63% of isolates. Only 3% of the isolates were negative for both genes [166]. The Stx encoding genes in *Aeromonas* are highly homologous to those of the most virulent strains of *E. coli*. In *Aeromonas,* these genes are thought to be present on plasmids, which tend to be lost during sub-cultivation, thus making them hard to detect [168]. At present, these genes have not been detected in foodborne isolates [102,166].

Adhesion to host surfaces is an essential initial step in most infections, and the ability to adhere has been regarded as an important virulence factor for many bacteria, including *Aeromonas* [169]. Motility is significant for attachment and the constitutive polar flagellum of *Aeromonas* spp. promotes colonization of surfaces, as demonstrated for *A. hydrophila*, *A. caviae* and *A. piscicola* [169,170,171]. In addition, multiple lateral flagella promoting swarming motility on more solid surfaces can be induced under some conditions [172]. The sequential actions of the latter two factors are hypothesized as key factors for the initial attachment of bacteria to the gastrointestinal epithelium or other body tissues, and for increased cell adherence, biofilm formation, and long-term colonization [172,173].

Undoubtedly, the virulence of *Aeromonas* spp. is complex and not fully understood, and the lack of definite virulence markers might contribute to an underestimation of the role of *Aeromonas* spp. in human disease. Following a diarrheal outbreak in Brazil, clinical and environmental isolates of *Aeromonas* spp. and *Vibrio cholerae* were investigated for the presence of known virulence genes. *Aeromonas* was identified as the sole pathogen in many diarrheic feces samples of patient, whereas the prevalence of *V. cholerae* was lower. Despite the higher prevalence of *Aeromonas* isolates in the clinical material, it was concluded that *V. cholerae* (O1) was the causative agent of the outbreak, mainly because of the ambiguous pattern of putative virulence genes in the isolated *Aeromonas* strains [157]. The mesophilic strains are gaining attention as important pathogens in humans due to improvements in methods for identification. However, no adequate animal model has yet been able to reproduce gastroenteritis caused by *Aeromonas* spp. [123], and the majority of studies concerning virulence have not assessed the expression of virulence genes nor secretion of toxins. Nevertheless, several in vitro and in vivo (animal model) experiments have attempted to elucidate the role of each virulence factor in the complex pathogenesis of *A. hydrophila* using mutant strains or purified toxins [174]. Clearly, reliable information about the true relevance of different *Aeromonas* spp. in various food matrices, especially in seafood, is missing at present. This is in light of the fact that minimally processed and RTE seafood are consumed by an increasing part of the population emphasizing the need for more knowledge about the actual microbiological risk associated with the presence of various *Aeromonas* species in seafood.

## 7. *Aeromonas* as a Spoilage Organism in Seafood

Microbial growth and metabolism resulting in the formation of amines, sulfides, alcohols, aldehydes, ketones and organic acids causing undesirable off-flavors are the main causes of food spoilage [175]. Various *Aeromonas* species is widely distributed in the aquatic environment and is commonly recognized as spoilage organisms in seafood, mainly in fish and crustaceans from tropical or warmer waters [11]. The spoilage potential of a particular microorganism is the result of the ability to produce the metabolites that are associated with the spoilage of a product [175], and the determination of the actual spoilage potential requires a combination of sensory, microbial and chemical analyses [176]. *A. salmonicida* (including psychrophilic strains responsible for causing the fish infection furuncluosis) is one of the most highlighted *Aeromonas* species regarding seafood spoilage. The species was identified as part of the spoilage microbiota in ice-stored sea bream (*Sparus aurata*) [177], Common carp [98], and cooked whole tropical shrimps (*Penaeus vannamei*) [178]. *Aeromonas*, *Pseudomonas* and *Enterococcus* were also identified as the dominating organisms in spoiled farmed shrimps (*Litopenaeus vannamei*) stored at 4 °C and 1 °C [179]. Shrimp incoculated against *A. salmonicida* was followed by storage under modified atmosphere produced off-odors described as sour, sulfur, and amine [178]. Strains of *A. salmonicida* isolated from Common carp produced mild off-odors characterized as “cheese and sour” and were able to reduce trimethylamine oxide (TMAO) to trimethylamine (TMA). *A. sobria* isolated from the same fish species were unable to produce off-odors or to reduce TMAO [98]. The spoilage potential of *A. sobria* in shrimp stored at 4 °C were reported by Yang et al. [180]. Moreover, *Aeromonas* spp. were identified as part of the dominating microbiota in spoiled grass carp (*Ctenopharyngodon idellus*) [181] and the species *A. allosaccharophila*, *A. eucrenophila* and *A. rivipollensis* were isolated from spoiled silver carp (*Hypophthalmichthys molitix*) fillet during storage at 4 °C [182]. A strain of *A. veronii* biovar *veronii* was characterized as a potential spoiler of fermented surimi as it produced a high level of total volatile basic nitrogen (TVB-N) and putrescine [176]. *Aeromonas* isolates associated with spoilage in fish and meat were characterized as slime producers, and 95% of the isolates showed lipase activity and protease activity [183]. Despite the recognition of various *Aeromonas* species as part of the microbiota in spoiled seafood products, there is little knowledge about the actual metabolite production resulting from the growth of *Aeromonas* species in different seafood products. Moreover, there is most likely differences in the spoilage potential at species and at strain level that need to be elucidated. High throughput sequencing (HTS) methods and metagenomic driven explorations of complex food-associated microbial consortia are numerous and have increased rapidly in recent years [184]. The culture-independent methods have contributed to a deeper understanding of the dynamics of the microbiota during food spoilage. However, there is need for more data on the role of various *Aeromonas* species as a spoilage organism in fish from cold water such as Atlantic salmon (*Salmo salar*), cod (*Gadus morhua*), and saithe (*Pollachius virens*). Studies of bacterial communities and important spoilage organisms under different production and storage regimes can help develop strategies for controlling food spoilage in perishable products.

## 8. The Prevalence and Growth of *Aeromonas* in RTE Seafood

Seafood products are more commonly contaminated by *Aeromonas* spp. than other food products, mainly because of the widespread occurrence of these bacteria in the marine environment [185]. *Aeromonas* spp. can enter the food chain through cross-contamination during processing from biofilms on food-contacted surfaces [186]. The food safety concern associated with the presence of *Aeromonas* spp. in seafood is a combination of the potential virulence of each strain and the ability to grow to disease-causing concentrations during storage. The occurrence of *Aeromonas* spp. in various habitats and their ability to persist and grow in continuously changing environments is exemplified with their viability over a relatively wide range of temperatures, pH and NaCl concentrations [187]. Environmental conditions may also affect the virulence and production of cytotoxic products, as demonstrated for *A. hydrophila* [188]. Moreover, strain variability must be accounted for as the ability of various *Aeromonas* strains to survive and grow under stressful conditions may wary between strains [189].

The optimal growth temperature for the majority of aeromonads is in the mesophilic range (28 to 30 °C). Many strains, especially clinical isolates, grow readily at temperatures up to 42 °C [190]. Most strains can survive and multiply at lower temperatures, from 2 to 10 °C, which highlights the need to monitor *Aeromonas* spp. in the cold chain [64,191]. This is especially the case for environmental isolates, and growth of *Aeromonas* spp. in food during refrigeration is commonly reported [192,193,194,195]. *Aeromonas* spp. were detected in relatively high concentrations in retail packages of rainbow trout (*Oncorhynchus mykiss*) (3.4 log CFU/g) and salmon (4.2 log CFU/g). The products were packed in trays wrapped with an oxygen-permeable film and the bacterial concentrations increased rapidly during refrigerated storage at 3 °C (>2 log units increase for four days) [196]. In retail sushi products with a shelf life of three days, the average concentration of *Aeromonas* spp. was 3 log CFU/g one day or more before the expiry date [19]. Moreover, the growth of mesophilic *A. salmonicida* in the magnitude of 1–4 log units within the shelf-life was demonstrated in a salmon-based sushi during cold storage [195].

Most studies concerning the growth kinetics of *Aeromonas* spp. in food during refrigeration were done in laboratory cultures and not performed as challenge studies in relevant food matrices. The majority of growth assessment studies comprise *A. hydrophila* in combination with various antimicrobial treatments [193,197,198] or with mixed *Aeromonas*-populations without species identification [194,199,200]. For example, growth of *A. hydrophila* during refrigerated storage was demonstrated for oysters [201], chicken breast [202], and squid (*Sepioteuthis sepioidea*) [193] in aerobic atmosphere. The same *A. hydrophila* strain was used in the two latter studies and substrate specific differences in growth kinetics were observed, as the specific growth rate was more than two times higher in chicken than in squid.

Species belonging to the genus *Aeromonas* are generally recognized as sensitive to low pH and the general assumption has been that *Aeromonas* do not represent a problem in food with pH < 6 [203]. Reduction of pH is an important and efficient action used by the food industry to stabilize food by inhibition of microbial growth, e.g., acidification of sushi rice (also decisive for the sensory properties). To evaluate the effect of pH as a hurdle against growth of *Aeromonas* in seafood products, the tolerability towards e.g., low pH must be decided on a species level. There seems to be a high heterogeneity in the ability of *Aeromonas* to grow in various conditions, and the ability is more strain than species dependent or related to the source of isolation. *Aeromonas* strains isolated from food have demonstrated the ability to grow in pH 4–5, and growth, although reduced, has been demonstrated in various low-pH seafood such as ceviche (raw fish and vegetables marinated in lime juice) (pH ≈ 5) [204], and in sushi (pH = 4.6 in the rice) [195]. In the latter study, heterogeneity in pH tolerance between strains of *A. salmonicida* and between *A. salmonicida* and other species was shown, especially in the low pH range, which is important for food processing. A practical issue related to growth prediction under various conditions is the limited knowledge on the interaction effect of low pH with low temperature and reduced water activity [205]. In brain heart infusion (BHI) medium at 28 °C, *A. hydrophila* can grow between pH 4.5 and 9.0 and 0–4% NaCl [190]. However, the combined effect of pH 5.3, 1.5% NaCl and a temperature of 5 °C did not support the growth of *A. hydrophila* in BHI [206]. No data exist for other *Aeromonas* species.

The trend of reduced food processing calls for more emphasis on the use of combined hurdles against microbial growth in addition to modified atmosphere packaging (MAP) combined with refrigerated storage. In general, *Aeromonas* spp. are not very sensitive to vacuum or MAP, and can grow relatively uninhibited when the competitive microbiota is low [200,207]. Thus, refrigerated modified atmosphere packaged seafood, including vacuum packages, could be an excellent niche for some *Aeromonas* spp. For instance, growth of *A. hydrophila* in sea bream fillets was demonstrated at 4 °C in 60/40% and 70/30% O_2_/N_2_, and the same strain was able to grow at 0 °C under air storage [191]. According to Qian and colleagues [208], *Aeromonas* spp. was predominant in fresh MAP shrimp microbiota but were inhibited during MAP refrigerated storage (80% CO_2_/15% N_2_, 5% O_2_). The growth of various *Aeromonas* species in seafood stored in modified atmosphere is not extensively studied, and more knowledge about substrate and strain heterogeneity is needed to apply efficient hurdles against these organisms.

Several guidelines are available for the assessment of the microbial quality of RTE food [209,210,211,212,213]. These guidelines include indicator organisms such as total aerobic bacterial count, Enterobacteriaceae and *E. coli*, and the pathogenic bacteria *S. aureus*, *B. cereus*, *V. parahaemolyticus*, *C. perfringens*, *Campylobacter* spp., and *Salmonella*. Regulatory limits exist for the presence of *L. monocytogenes* in RTE food [214]. At present, *Aeromonas* is not included in any guideline on the microbiological quality of RTE food. An increasing part of the RTE food on the market is based on raw seafood combined with raw vegetables. Given the widespread occurrence of *Aeromonas* spp. in these ingredients, quality and food safety assessment of, e.g., stored retail sushi and sashimi grade fish, should include quantification of *Aeromonas* spp. A remaining issue is, however, how to distinguish the truly virulent strains from those who will not likely cause illness. A guidance limit of 3–5 log CFU/g in food products for raw consumption might be proposed based on the reported concentrations of *Aeromonas* spp. in food from various outbreaks and the ability of various strains to grow under several time-temperature-atmosphere scenarios (cited throughout the present work).

## 9. Conclusions and Perspectives

Undoubtedly, fish and seafood are beneficial for human health, but the consumption of minimally processed food is accompanied by food safety challenges. The genus *Aeromonas* has been recognized as an “emerging foodborne pathogen” for many years, but we still do not know how to identify the truly virulent strains.

RTE seafood products intended for consumption without heat treatment should be treated as risk products for *Aeromonas*-mediated illness because of (1) the ubiquitous presence of *Aeromonas* spp. in seafood, (2) the high prevalence of *Aeromonas* spp. in vegetables that can constitute various RTE seafood meals, (3) the potential for cross-contamination between assembled ingredients in combined products, which might lead to enhanced growth conditions, (4) the ability of *Aeromonas* to grow during cold storage, which can support growth to disease-causing concentrations, and (5) these products are eaten by an increasing amount of people in the risk groups, such as children and the elderly. The presence of *Aeromonas* spp. in various spoiled seafood products highlights the role of these bacteria as spoiler organisms. The ongoing evolution in the culture-independent approach to food microbiology can potentially contribute to a deeper understanding of the microbial transformations during complex processes such as bacterial spoilage. Such knowledge can be applied to develop strategies for controlling food spoilage in perishable products. Hurdles against growth of strains with spoilage or disease potential must be applied to prevent growth in refrigerated, vacuum and modified atmosphere packaged food.

In light of the previous taxonomic controversies of the genus *Aeromonas*, the frequent species reclassifications, the lack of definite virulence markers and the problem with ambiguous biochemical tests, there is still a need for characterization of circulating clinical and environmental strains to provide insight into the role of these bacteria as foodborne pathogens. Another remaining issue is to establish which species are the most clinically relevant and which factors are decisive for pathogenicity. Whole genome sequencing is a potential game changer in providing identification and characterization of pathogenic bacteria for epidemiological investigations [215]. Retrieving data on pathogen genomes directly from metagenomic data provides a possibility to identify unknown or overlooked etiological organisms that cause millions of cases of foodborne illness each year [57,216,217]. Future studies on *Aeromonas* strains from seafood products should aim to couple systematized and validated data on the distribution of virulence factors with relevant HTS data, in particular whole-genome sequences of highly virulent strains. Such data are still missing.

## Figures and Tables

**Table 1:** Biological hazards associated with seafood (adapted and modified from [11]).

Type of Illness	Causative Agent
Bacterial infections	*Aeromonas* spp., *Bacillus cereus, Campylobacter* spp., *Clostridium perfringens, Escherichia coli*, *Listeria monocytogenes*, *Salmonella* spp., *Shigella* spp., *Vibrio* spp. (*V. parahaemolyticus*, *V. cholerae*, *V. vulnificus*)
Bacterial intoxications	*Clostridium botulinum*, *Staphylococcus aureus*
Viral infections	Hepatitis A, Norovirus
Parasitic infections	Nematodes (round worms), cestodes (tapeworms), trematodes (flukes)
Intoxication by biotoxins	Ciguatera, paralytic shellfish poisoning (PSP), diarrheic shellfish poisoning (DSP), amnesic shellfish poisoning (ASP), neurotoxic shellfish poisoning (NSP), histamine

**Table 2:** Currently recognized species belonging to the genus *Aeromonas* and the source of isolation of the type strain. The species in bold represent the most prevalent clinical species and lowercase superscript letters indicate that the species have been isolated from clinical species (a), water (b), and fish/seafood (c) (The table is adapted and modified from [64]).

Species	Source of Isolation (Year) of Type Strain	Reference
***A. allosaccharophila*** ^a,c^	Eel (1992)	[65]
*A. aquatica* ^b^	Lake water (2015)	[66]
*A. aquatilis* ^b^	Lake water (2017)	[63]
*A. australiensis* ^b^	Water (2013)	[67]
*A. bestiarum* ^a–c^	Diseased fish (1996)	[68]
*A. bivalvium* ^c^	Bivalve mollusks (2007)	[69]
*A. cavernicola* ^b^	Fresh water (2013)	[70]
***A. caviae*** ^a–c^^,†^	Guinea pig (1984)	[71]
*A. crassostreae* ^c^	Oyster (2017)	[63]
***A. dhakensis*** ^a–c,*^	Aquarium water (2008)/Human (diarrheic stool) (2002)	[72,73,74]
*A. diversa* ^a^	Human (wound infection) (2010)	[61]
*A. encheleia* ^a,c^	Eel (1995)	[75]
*A. enterica* ^a^	Human (diarrheic stool) (2017)	[63]
*A. eurenophila* ^a–c^	Fresh water fish (1998)	[76]
*A. finlandiensis* ^b^	Lake water (2015)	[66]
*A. fluvialis* ^b^	River water (2010)	[77]
***A. hydrophila*** ^a–c^	Milk (1943)	[78]
***A. intestinalis*** ^a^	Human (diarrheic stool) (2017)	[63]
***A. jandaei*** ^a–c^	Human (feces) (1991)	[79]
*A. lacus* ^b^	Lake water (2015)	[66]
*A. lusitana* ^b^	Water (2012)	[62]
***A. media*** ^a–c^	Water (1983)	[80]
*A. molluscorum* ^c^	Bivalve mollusks (2004)	[81]
*A. piscicola* ^c^	Diseased fish (salmon) (2009)	[82]
*A. popoffii* ^a,b^	Drinking water (1997)	[83]
*A. rivipollensis* ^b^	River sediments (2016)	[84]
*A. rivuli* ^b^	Water rivulet (2011)	[85]
*A. salmonicida* ^a–c,**^	Fish (salmon) (1953)	[86]
*A. sanarellii* ^a^	Human (wound infection) (2010)	[87]
*A. schubertii* ^a,c^	Human (1988)	[88]
*A. simiae*	Monkey feces (2004)	[89]
*A. sobria* ^a–c^	Fish (1976)	[90]
*A. taiwanesis* ^a^	Human (wound infection) (2010)	[87]
*A. tecta* ^a,c^	Human feces (2008)	[91]
***A. trota*** ^a,^^††^	Human feces (1991)	[92]
***A. veronii*** ^a–c^	Human (sputum) (1987)	[93]

^†^ Synonymous with *A. punctate*; * *A. aquariorum* and *A. hydrophila* subsp. *dhakensis* were synonymized under the name *A. dhakensis*; ** The subset of mesophilic, motile strains that can grow at 37 °C; ^††^ Synonymous with *A. enteropelogenes*.
